# Cultivable Gut Microbiota in Synanthropic Bats: Shifts of Its Composition and Diversity Associated with Hibernation

**DOI:** 10.3390/ani13233658

**Published:** 2023-11-26

**Authors:** Igor V. Popov, Iraida S. Berezinskaia, Ilia V. Popov, Irina B. Martiusheva, Elizaveta V. Tkacheva, Vladislav E. Gorobets, Iuliia A. Tikhmeneva, Anna V. Aleshukina, Tatiana I. Tverdokhlebova, Michael L. Chikindas, Koen Venema, Alexey M. Ermakov

**Affiliations:** 1 Faculty “Bioengineering and Veterinary Medicine” and Center for Agrobiotechnology, Don State Technical University, 344000 Rostov-on-Don, Russia; ivpopov@donstu.ru (I.V.P.); etkacheva@donstu.ru (E.V.T.); waldemar1866@gmail.com (V.E.G.); juliya5634@gmail.com (I.A.T.); tchikind@sebs.rutgers.edu (M.L.C.); amermakov@yandex.ru (A.M.E.); 2 Division of Immunobiology and Biomedicine, Center of Genetics and Life Sciences, Sirius University of Science and Technology, 354340 Federal Territory Sirius, Russia; 3 Centre for Healthy Eating & Food Innovation (HEFI), Maastricht University—Campus Venlo, 5928 SZ Venlo, The Netherlands; k.venema@maastrichtuniversity.nl; 4 Rostov Research Institute of Microbiology and Parasitology, 344010 Rostov-on-Don, Russia; aleshukina@yandex.ru (I.S.B.); ibmartyusheva@mail.ru (I.B.M.); aaleshukina@mail.ru (A.V.A.);; 5 Health Promoting Naturals Laboratory, School of Environmental and Biological Sciences, Rutgers State University, New Brunswick, NJ 08901, USA; 6 Department of General Hygiene, I.M. Sechenov First Moscow State Medical University, 119435 Moscow, Russia

**Keywords:** bats, gut microbiota, hibernation, microbial diversity

## Abstract

**Simple Summary:**

Bats play a significant role in public health and animal welfare as these animals are hosts for emerging pathogens. Urbanization has resulted in the acquisition of these animals’ synanthropic behavior. Urban infrastructure suits bats as their hibernacula more than their natural sites due to the absence of predators, relatively high temperatures, and the close location of potential roost sites. However, the microbiota of hibernating bats is little studied, leaving some degree of uncertainty on how the abundance and diversity of gut bacteria are affected by low body temperature and food deprivation. In this study, we investigated the composition of cultivable gut microbiota before, during, and after hibernation of bats in the rehabilitation center. As a result, we observed a high abundance of opportunistic pathogens in the gut microbiota of active bats that had just arrived at the rehabilitation center that they had probably received from the environment. During the hibernation of bats, the abundance of these bacteria was significantly decreased, while the abundance of some other commensals was increased with an overall decrease in microbial diversity. This study shows how hibernation affects bat gut microbiota, pointing to the necessity of investigation of the bat–microbiota relationship during hibernation to prevent emerging diseases.

**Abstract:**

The role of bats in the global microbial ecology no doubt is significant due to their unique immune responses, ability to fly, and long lifespan, all contributing to pathogen spread. Some of these animals hibernate during winter, which results in the altering of their physiology. However, gut microbiota shifts during hibernation is little studied. In this research, we studied cultivable gut microbiota composition and diversity of *Nyctalus noctula* before, during, and after hibernation in a bat rehabilitation center. Gut microorganisms were isolated on a broad spectrum of culture media, counted, and identified with mass spectrometry. Linear modeling was used to investigate associations between microorganism abundance and *N. noctula* physiological status, and alpha- and beta-diversity indexes were used to explore diversity changes. As a result, most notable changes were observed in *Serratia liquefaciens*, *Hafnia alvei*, *Staphylococcus sciuri*, and *Staphylococcus xylosus*, which were significantly more highly abundant in hibernating bats, while *Citrobacter freundii*, *Klebsiella oxytoca*, *Providencia rettgeri*, *Citrobacter braakii*, and *Pedicoccus pentosaceus* were more abundant in active bats before hibernation. The alpha-diversity was the lowest in hibernating bats, while the beta-diversity differed significantly among all studied periods. Overall, this study shows that hibernation contributes to changes in bat cultivable gut microbiota composition and diversity.

## 1. Introduction

Bats play a significant role in global microbial ecology as their ability to fly and their long lifespan in comparison with other mammals of the same size contribute to a wider spread of emerging pathogens [1]. The evolutionary acquisition of the ability to fly has resulted in boosting energy expenditure and metabolism rates in bats, which in turn has resulted in obtaining unique immune system features, such as a limited inflammatory response against pathogens [2]. Also, recent comparative genomic studies have shown that unique immune system features could be associated with the loss of some genes that are responsible for regulating immune responses in mammals [3,4]. All of these features contribute to distinctive host–microbiome interactions, which in turn make bats “living bioreactors” for emerging diseases as they can asymptomatically carry pathogens for a long time [5,6,7].

Some bats, insectivorous ones in particular, hibernate during winter [8]. Hibernation is an adaptational process for seasonal cycles of feeding and fasting and is intended to maintain the integrity of organ systems when dietary nutrients are lacking [9]. Food deprivation and a decrease in body temperature directly affect the host’s microbiota composition as the conditions for microbial fermentation become less favorable [10]. As a result, the microbiota during the hibernation of animals is less diverse in comparison with their active state [11,12,13].

Urbanization has significantly contributed to the changing ecology of bats, as the expansion of urban and agricultural infrastructure forces bats to syntropy as their natural sites for roosting are shrinking [14,15]. As a result, a lot of bat colonies are often found in households as there are few places in cities where bats can safely roost and hibernate [15,16,17]. Knowing that, during winter, bats are in direct or indirect contact with humans and domestic animals and that hibernation could alter the microbiota composition and diversity, it is important to study the bat microbiota during their life cycle to predict possible interspecies transmissions of microbes.

There are few studies published on gut microbiota composition and diversity of bats during their hibernation. Recent studies are dedicated to *Rhinolophus ferrumequinum* and *Rhinolophus pusillus* from Jiyuan City, China [18,19]. In this study, we provide data on the cultivable gut microbiota composition and diversity shifts before, during, and after *Nyctalus noctula* hibernation in a bat rehabilitation center in Rostov-on-Don, Russia.

## 2. Materials and Methods

### 2.1. Sampling

The *N. noctula* colony used was seized by Don State Technical University bat rehabilitation center volunteers in November 2022 from a household at the request of the owners. Then, the colony was transferred to the rehabilitation center, and, after veterinary examination, 13 clinically healthy bats were selected for separate keeping. The bats were kept collectively in one box to ensure socialization during hibernation; their diet while in the rehabilitation center consisted of superworms and mealworms. Bats were put into hibernation in late November 2022 at a constant temperature of 10 °C and brought out in early April 2023. To assess bat gut microbiota community change before, during, and after hibernation, a minimum of 0.5 g of pooled fecal samples (*n*) was taken from bats in their active state in November 2022 (*n* = 13), during their hibernation in March 2023 (*n* = 19), and in their active state two weeks after awakening from hibernation in April 2023 before the release of these animals to wildlife (*n* = 13). Samples were placed in sterile tubes and immediately transported to the laboratory at 7 °C.

### 2.2. Gut Bacteria Isolation, Counting, and Identification

Samples were aseptically removed from tubes for subsequent isolation by vortexing for 1 min with sterile glass beads in sterile phosphate-buffered saline (pH 7.4) at a ratio of 1:10. Then, the suspensions were 10-fold serially diluted and studied by a qualitative–quantitative method using the following differential diagnostic solid nutrient media: blood agar for hemolytic bacteria isolation; yolk–salt agar for staphylococci isolation, Sabouraud agar with antibiotics for the *Candida* isolation; Endo agar, Bismuth sulfite agar, and Ploskirev agar for enterobacteria isolation; Schaedler agar for the anaerobic bacteria isolation, including coryneform bacteria; Uriselect 4 agar for the ESCAPE bacteria isolation and differentiation; MRS agar for lactic acid bacteria isolation; Wilson Blair agar for the clostridia isolation; and semi-liquid Bifidum medium for bifidobacterial isolation. The cultures were incubated in a microaerophilic chamber at 37 °C for 24–48 h. The selection of colonies for further studies was carried out based on colony morphology and microscopy with Gram staining. The number of colony forming units per gram of fecal sample (CFU/g) was counted from the dilution of 30–300 colonies, multiplied by the respective dilution.

Microflex benchtop mass spectrometer with a MALDI Biotyper database and FlexControl software (https://www.bruker.com/en.html, Leipzig, Germany) was used for the identification of bacterial species. To obtain pure bacterial cultures from diagnostic media for mass spectrometric identification, we used the Givental–Vedmina agar (GVA) medium. Sample preparation for the study of pure cultures of strains was carried out according to the manufacturer’s protocol. Briefly, daily pure cultures of bacteria in the form of single colonies were applied in a thin layer directly to the target point, starting from the middle. Target dots coated with biomaterial were coated with 1 μL of HCCA (α-cyano-4-hydroxycinnamic acid) matrix solution and allowed to dry completely at room temperature. After drying, the target with cultures was placed into a mass spectrometer. The level of identification of bacteria was interpreted according to the score criteria specified in the instructions: 2.300–3.000 meant a high probability of species identification; 2.000–2.299 meant reliable genus identification and probable species identification; 1700–1999 meant probable genus identification; 0.00–1.699 meant unreliable identification. As a result, we obtained a database with identified isolates and their abundance in the form of colony-forming units per gram (CFU/g), which we used for further statistical analyses.

### 2.3. Statistical Analyses

The programming language R v4.2.3 (R Foundation for Statistical Computing, Vienna, Austria) was used for statistical analyses. The “vegan” package was used for the calculation of alpha- and beta-diversity indexes [20]. For the comparison of the alpha-diversity of cultivable gut microbiota of studied bats, we used the Shannon index to assess the entropy of bacterial communities, the Chao1 index to assess the richness of bacterial species based on their abundance, and the Pielou index to assess the evenness of studied microbial community. For the beta-diversity, we used the Bray–Curtis index to analyze dissimilarity in bacterial species abundance in bat fecal samples before, during, and after their hibernation. The Kruskal–Wallis test was performed to determine differences in the alpha-diversity indexes, and pairwise multiple comparisons were performed with the Mann–Whitney test. PERMANOVA with the adonis function from the vegan package was used to determine differences in beta diversity distances (the number of permutations was set to 1000). The second version of the “Microbiome Multivariable Association with Linear Models” package (MaAsLin2) was used for differential abundance analysis based on CFU/g count data [21]. Before differential abundance analysis, we performed total sum scaling normalization of the count data. Microbial composition during hibernation was used as a reference for the linear model analysis. All multiple comparison results were adjusted with the Benjamini–Hochberg false discovery rate. Adjusted *p*-values (*q*-values) were considered significant at *q* < 0.05. Results were visualized with the “ggplot2” package [22].

## 3. Results

### 3.1. Cultivable Gut Microbiota Diversity

**Figure 1:**
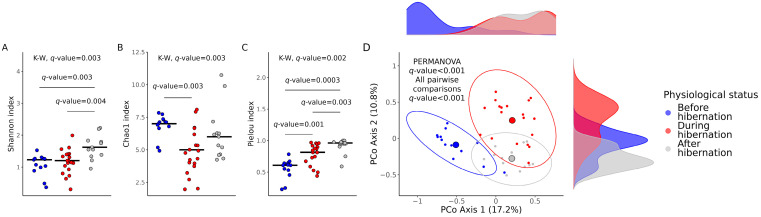
Alpha- and beta-diversity metrics in fecal samples obtained before, during, and after hibernation of Nyctalus noctula. Jitter plots show differences in alpha-diversity based on (**A**) Shannon index, (**B**) Chao1 index, and (**C**) Pielou’s evenness. Principal coordinate analysis (PCoA) plot (**D**) illustrates differences in beta-diversity based on the Bray–Curtis distance matrix. *Q*-values for alpha-diversity indexes were calculated with the Benjamini–Hochberg adjustment for the Kruskal–Wallis overall comparison and for the Mann–Whitney test for pairwise comparisons. *Q*-values for beta-diversity differences were calculated with the PERMANOVA test following the Benjamini–Hochberg adjustment for pairwise comparisons; the number of permutations was set to 1000.

Alpha-diversity indexes of gut microbial communities differed significantly among bats with different physiological statuses. The Shannon index was significantly higher in the gut microbiota of bats after waking up from hibernation in comparison with bats before and during hibernation. The Chao1 index was significantly decreased in hibernating bats compared with bats before hibernation. On the contrary, the lowest values in Pielou’s index were observed in samples acquired from bats before hibernation; yet, the evenness of gut microbiota in bats during hibernation was significantly lower in comparison with bats after waking up (Figure 1A–C).

The beta-diversity of cultivable gut microbiota communities based on the Bray–Curtis dissimilarity index also differed significantly in bats before, during, and after their hibernation (*q* < 0.001). Values from bats that woke up from hibernation took an intermediate position between the values from bats before and during their hibernation in the calculated beta-diversity matrix, as seen in the principal coordinate analysis plot (Figure 1D).

### 3.2. Cultivable Gut Microbiota Composition

**Figure 2:**
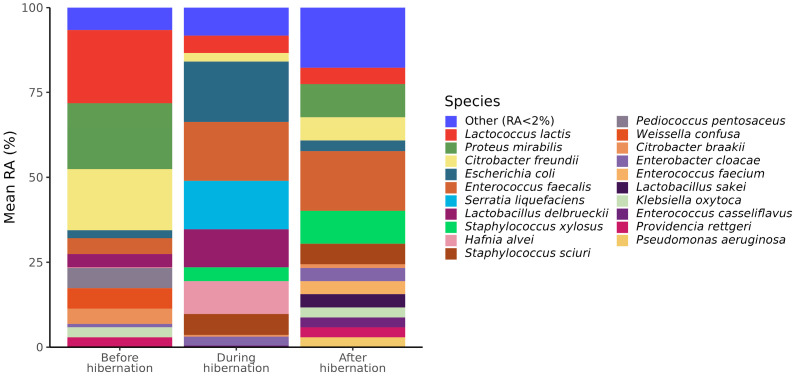
Stacked bar charts representing cultivable gut microbiota composition (as relative abundance (RA) of bacterial species) in fecal samples obtained before, during, and after the hibernation of *Nyctalus noctula*. Taxa in the legends are ordered from the most abundant to the least among all three studied groups. The category “other” contains species that mean RA among all groups less than 2%.

The most abundant bacterial species in the gut microbiota of bats before hibernation were *Lactococcus lactis* (21.6%), *Proteus mirabilis* (19.4%), and *Citrobacter freundii* (18%). *Escherichia coli* (17.8%), *Enterococcus faecalis* (17.4%), *Serratia liquefaciens* (14.3%), and *Lactobacillus delbrueckii* (11.1%) were among the most abundant bacteria observed in the gut microbiota of hibernating bats. The most abundant bacterial species in the gut microbiota of bats after hibernation was *Enterococcus faecalis* (17.5%), while the abundance of other bacteria did not exceed 10%, which indicates higher evenness of bacterial abundance related to bats of other physiological states, which was also observed in the difference in Pielou’s index. The gut microbiota composition of bats with different physiological statuses related to hibernation is shown in Figure 2 and Figure S1.

**Figure 3:**
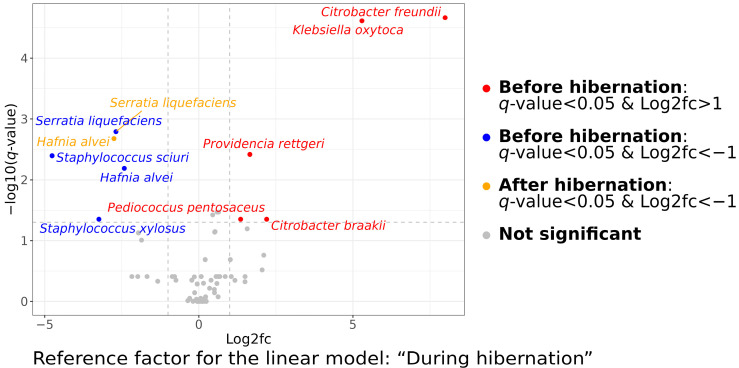
Volcano plot illustrating the results of the differential abundance analysis of bacterial species isolated from fecal samples of *Nyctalus noctula* before, during, and after hibernation. Microbial composition during hibernation was used as a reference for the linear model analysis. Blue, yellow, and red dots on the plot show bacteria, the abundance of which had significant associations with studied physiological statuses related to hibernation. Blue dots represent bacteria whose abundance was significantly lower in bats before hibernation in comparison with the reference. Yellow dots represent bacteria whose abundance was significantly lower in bats after hibernation in comparison with the reference. Red dots represent bacteria whose abundance was significantly higher in bats before hibernation in comparison with the reference.

**Figure 4:**
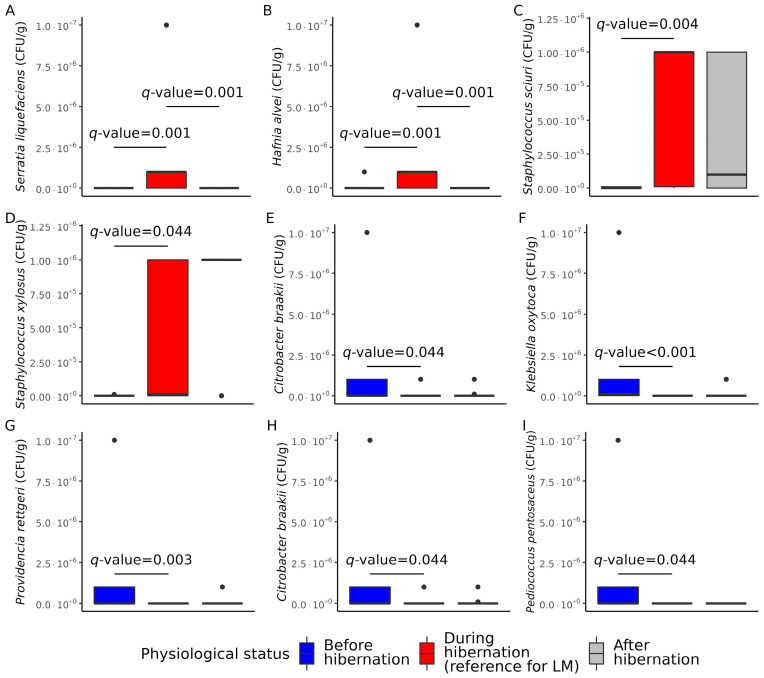
Box plots illustrating the abundance of identified species in the form of colony-forming units per gram (CFU/g) that differ significantly in active bats before and after hibernation in comparison with hibernating bats: (**A**) *Serratia liquefaciens*, (**B**) *Hafnia alvei*, (**C**) *Staphylococcus sciuri*, (**D**) *Staphylococcus xylosus*, (**E**) *Citrobacter braakii*, (**F**) *Klebsiella oxytoca*, (**G**) *Providencia rettgeri*, (**H**) *Citrobacter braakii*, and (**I**) *Pedicoccus pentosaceus*. *Q*-values were calculated with MaAsLin2 linear model (LM) analysis following Benjamini–Hochberg false discovery rate correction.

After performing differential abundance analysis, we found that an abundance of nine species was significantly different among studied physiological statuses relative to hibernation. These bacteria were *Serratia liquefaciens* (*q*-value = 0.001), *Hafnia alvei* (*q*-value = 0.006), *Staphylococcus sciuri* (*q*-value = 0.004), and *Staphylococcus xylosus* (*q*-value = 0.044), which were significantly more highly abundant in hibernating bats, while *Citrobacter freundii* (*q*-value = 0.00002), *Klebsiella oxytoca* (*q*-value = 0.00002), *Providencia rettgeri* (*q*-value = 0.003), *Citrobacter braakii* (*q*-value = 0.044), and *Pedicoccus pentosaceus* (*q*-value = 0.044) were significantly more abundant in active bats before the hibernation (Figure 3 and Figure 4). The results of differential abundance analysis on the genus, family, and order levels are shown in Supplementary Figures S2, S3, S6, and S7. There were no significant differences in the class and phylum levels.

## 4. Discussion

In this study, we investigated the cultivable gut microbiota composition and alpha/beta diversity of *N. noctula* bats before, during, and after their hibernation in the bat rehabilitation center in Rostov-on-Don and made a statistical comparison of these features. Currently, *N. noctula* is one of the most common bat species in the Rostov region according to the observations of the DSTU bat rehabilitation center. This species is prone to synanthropy. Their body size and ability to fly allow them to roost in city/town buildings in places difficult for humans to reach [23,24]. However, bats living as close neighbors within urban areas may be unwanted for citizens, which is why a lot of people tend to destroy whole bat colonies when they find them in households, which poses a great risk for these animals. That is why there are several bat rehabilitation centers around the world, whose primary aim is to save bat populations during winter [25,26]. At the same time, bat rehabilitation centers provide a unique opportunity to study pathogen surveillance in these animals [27,28]. This opportunity is especially important for detecting potential progenitors of emerging viruses as bats are recognized as natural hosts for many viral human pathogens [29,30,31,32,33]. However, it is worth noting that the viral microbiota of bats is significantly more studied than the bacterial microbiota. Because bats are in close contact with humans and the immune system of bats allows them to co-exist with pathogens due to the inhibition of inflammatory responses, we strongly believe that the bacterial microbiota of bats should also be studied as it could be the missing link in emerging diseases that originate from bats [5,34]. Especially, it is important to study the bats’ hibernative bacterial microbiota, as the alterations in bacterial composition shifts related to hibernation are less studied, and, during this time, bats could be in the closest contact with humans and domestic animals.

There are few studies published on microbiota alterations during the hibernation of bats. One of the major topics of scientific interest is studying the fungal microbiota of bats, as fungi, *Pseudogymnoascus destructans* in particular, are microorganisms that pose the greatest danger to bats due to the lack of adaptational immune responses against them during hibernation. The white-nose syndrome (WNS), caused by *P. destructans*, results in a dramatic decrease in bat populations [35,36,37]. Interestingly, the microbiota of hibernating bats could protect their hosts from fungal infections. Lemieux-Labonté et al. studied skin microbiota composition and diversity of hibernating WNS-positive and WNS-negative bats and found that the alpha-diversity of WNS-positive bats was significantly lower than in WNS-negative bats, which was caused by the significant enrichment of *Janthinobacterium*, *Micrococcaceae*, *Pseudomonas*, *Ralstonia*, and *Rhodococcus* abundance. These bacteria, *Rhodococcus* and *Pseudomonas* in particular, can contribute to the resistance of bats to *P. destructans*. They could inhibit other taxa to increase their own abundance in skin microbiota, resulting in an alpha-diversity decrease [38]. Later, Lemieux-Labonté et al. conducted controlled experiments of WNS modeling in bats in lab conditions and found a significant increase in the relative abundance of *Rhodococcus* and *Pseudomonas* in WNS-positive bats [39].

Apart from skin microbiota, gut microbiota alterations during the hibernation of bats have also been studied. Xiao et al. studied gut microbiota composition and diversity of *Rhinolophus ferrumequinum* in different seasonal periods (early spring, early summer, and late summer), and found that the alpha-diversity in hibernating bats was significantly decreased in comparison with bats before their hibernation. Also, the relative abundance of *Pseudomonas* was significantly higher in hibernating bats in comparison with bats before hibernation, while the relative abundance of *Enterococcus* was significantly lower. On the phylum level, the relative abundance of *Firmicutes* was significantly lower in bats during hibernation in comparison with before hibernation. It should be noted that, in this study, the microbial composition of bats after waking up from hibernation (early spring) had less significant differences to bats during their hibernation than in bats before the hibernation, which points to the fact that the gut microbiota of bats could slowly restore from the hibernation of their host and this process could be related to the abundance of available food in the environment [18]. In another study, Liu et al. examined the gut microbiota composition of the same bat species in their active state in summer and in winter during their hibernation, but at two different locations with different temperatures during winter: 11.6 °C to 16.8 °C in a canal tunnel and 17.1 °C to 18.0 °C in a mine tunnel. As a result, they not only found that there were significant differences in microbiota composition between the hibernating and active bats but also between bats with two different hibernacula sites. The alpha-diversity of gut microbiota in bats hibernating in the mine tunnel was significantly lower than in bats hibernating in the canal tunnel, where the temperature was lower. Also, the differences in ambient temperature in hibernacula resulted in marked site-specific differences in the relative abundances of Firmicutes, Actinobacteria, and Tenericutes [19]. It should also be noted that, in the two above-mentioned studies, authors conducted a differential abundance analysis of predicted functional microbiota pathways based on 16S rRNA metagenomic sequencing data and determined that the gut microbiota metabolism could be converted from carbohydrate-related to lipid-related in hibernating bats [18,19].

Our study was conducted in the conditions of a rehabilitation center, which could have influenced the microbiota of bats. However, we observed a decrease in the alpha-diversity of bat gut microbiota communities, which corresponded to the results of the above-discussed reports. We also observed significant differences in beta-diversity, where the most prominent distances were between the gut microbiota communities of bats before and during hibernation, while the beta-diversity values of the intestinal microbiota of bats after waking up from hibernation had intermediate positions in a distance matrix between the two other studied physiological statuses. In addition to this, the differences in the abundance of bacterial species were also the most evident between the gut microbiota of bats before and during hibernation, while differences in bats after waking up from hibernation were less prominent, although, as observed by Xiao et al. [18], there was a tendency to return to the before-hibernation composition. We speculate that the gut microbiota in bats after hibernation had not yet fully returned to the state before the hibernation as the time of sampling was only two weeks after their waking up. Nevertheless, the alpha-diversity indexes were significantly higher in bats after waking up in comparison with their hibernating state. This can be explained by the fact that all the bats included in the study were fattened before the final sampling, which is essential before the release of the animals to the wildlife after rehabilitation, while, in other studies, free-roaming bats on food self-sufficiency were studied [18,19]. In our study, the abundance of *S. liquefaciens* and *H. alvei* was significantly higher in hibernating bats compared with bats in active states. These bacteria are recognized as opportunistic pathogens in humans and animals and previously have been observed in the gut microbiota of *Myotis myotis* and *Rhinolophus hipposideros* in Slovakia [40,41,42]. *S. sciuri* and *S. xylosus*, which are also significantly more abundant in hibernating bats, are commensal animal-associated bacteria that could be involved in the spreading of antibiotic-resistance and virulence genes among domestic and farm animal microbiota according to recent reports [43,44,45]. In the gut microbiota of active bats before their hibernation, *C. freundii*, *K. oxytoca*, *P. rettgeri*, *C. braakii*, and *P. pentosaceus* were significantly more abundant in comparison with hibernating bats. Most of them, except *P. pentosaceus*, are opportunistic pathogens, which cause nosocomial infections in humans [46,47,48,49]. Fecal samples from bats before their hibernation were acquired right after their arrival at the rehabilitation center, which indicated that bats could acquire these bacteria from the environment. *C. freundii*, *K. oxytoca*, *P. rettgeri*, and *C. braakii* are commonly found in water, soil, and food [50,51,52,53]. It is a well-known fact that the gut microbiota of bats strongly depends on the environment, as their gastrointestinal tract is relatively shorter than in animals of the same size, which is the result of their evolutionary adaptation for flight [54,55,56]. So, our study provides additional evidence for the dependence of bat microbiota on the environmental microbiome, although the fundamental mechanisms of host–microbiota interactions that allow bats to co-exist with opportunistic pathogens should be discovered. There was *P. pentosaceus* among the bacterial species, with a high abundance in active bats before hibernation. *P. pentosaceus* is a lactic acid bacterium that is gradually attracting attention as a potential probiotic [57]. In one of our previous studies, we searched for probiotics in *N. noctula* gut microbiota, but most of the studied strains occurred to be pro-mutagenic, making them unsafe candidates [5]. However, it does not mean that the microbiota of bats could not be the site for the isolation of new probiotics. Unique microbiota features of bats could provide new solutions against emerging pathogens. For example, the above-discussed studies by Lemieux-Labonté et al. could be the basis for searching for new probiotics with strong antifungal effects [38,39,58].

In this study, we investigated the gut microbiota composition and diversity of *N. noctula* by isolating microorganisms on the broad spectrum of microbiological media combined with taxonomical identification with mass-spectrometry. Nevertheless, we should mention that non-culture methods, such as high-throughput sequencing of 16S rRNA amplicons or the whole metagenomic DNA, could have provided more detailed data, as this method allows the investigation of uncultivable microbiota resulting in a broader range of identified taxa for following composition and diversity analysis [59]. However, in our study, isolation and culturing of gut bacteria on the broad spectrum of microbiological media allowed us to conduct a comprehensive diversity analysis, the results of which correspond to other studies, and, most importantly, the counting of bacterial composition in the form of CFU/g allowed us to perform statistical analysis of absolute abundance data, while statistical analysis of metagenomic sequencing requires other approaches to proceed relative abundance data, which could result in false positives and irreproducibility of results among different reports [60,61].

## 5. Conclusions

This study shows the first data on cultivable gut microbiota composition and diversity of *N. noctula* before, during, and after their hibernation in the bat rehabilitation center in Rostov-on-Don. The diversity of cultivable gut microbiota of the studied bats in hibernation was significantly lower in comparison with bats in their active state. Hibernation resulted in shifts in the abundance of some bacteria, which are recognized as opportunistic pathogens for humans and domestic animals, indicating the importance of studying the bacterial microbiota of bats to prevent emerging infections.

## Data Availability

The raw data are available from corresponding authors upon a reasonable request.

## Supplementary Materials

The following supporting information can be downloaded at: https://www.mdpi.com/article/10.3390/ani13233658/s1, Figure S1. Stacked bar charts representing cultivable gut microbiota composition (as relative abundance (RA) of bacterial taxa) in fecal samples obtained before, during, and after the hibernation of *Nyctalus noctula*. RA at (A) phylum, (B) class, and (C) order levels. Taxa in the legends are ordered from the most abundant to the least among all three studied groups. Figure S2. Volcano plot illustrating the results of the differential abundance analysis of bacterial families isolated from fecal samples of *Nyctalus noctula* before, during, and after hibernation. Microbial composition during hibernation was used as a reference for the linear model analysis. Blue, yellow, and red dots on the plot show bacteria, the abundance of which had significant associations with studied physiological statuses related to hibernation. Blue dots represent bacteria whose abundance was significantly lower in bats before hibernation in comparison with the reference. Yellow dots represent bacteria whose abundance was significantly lower in bats after hibernation in comparison with the reference. Red dots represent bacteria whose abundance was significantly higher in bats before hibernation in comparison with the reference. Figure S3. Volcano plot illustrating the results of the differential abundance analysis of bacterial orders isolated from fecal samples of *Nyctalus noctula* before, during, and after hibernation. Microbial composition during hibernation was used as a reference for the linear model analysis. Blue and red dots on the plot show bacteria, the abundance of which had significant associations with studied physiological statuses related to hibernation. Blue dots represent bacteria whose abundance was significantly lower in bats before hibernation in comparison with the reference. Red dots represent bacteria whose abundance was significantly higher in bats before hibernation in comparison with the reference. Figure S4. Box plots illustrating the abundance of identified species in the form of colony-forming units per gram (CFU/g) that differ significantly in active bats before and after hibernation in comparison with hibernating bats: (A) *Serratia liquefaciens*, (B) *Hafnia alvei*, (C) *Staphylococcus sciuri*, (D) *Staphylococcus xylosus*, (E) *Citrobacter braakii*, (F) *Klebsiella oxytoca*, (G) *Providencia rettgeri*, (H) *Citrobacter braakii*, and (I) *Pedicoccus pentosaceus*. *Q*-values were calculated with MaAsLin2 linear model (LM) analysis following Benjamini–Hochberg false discovery rate correction. Figure S5. Box plots illustrating the abundance of identified genera in the form of colony-forming units per gram (CFU/g) that differ significantly in active bats before and after hibernation in comparison with hibernating bats: (A) *Staphylococcus*, (B) *Serratia*, (C) *Hafnia*, (D) *Citrobacter*, (E) *Klebsiella*, (F) *Proteus*, (G) *Providencia*, (H) *Pediococcus*, (I) *Pseudomonas*, and (J) *Weisella*. *Q*-values were calculated with MaAsLin2 linear model (LM) analysis following Benjamini–Hochberg false discovery rate correction. Figure S6. Box plots illustrating the abundance of identified families in the form of colony-forming units per gram (CFU/g) that differ significantly in active bats before and after hibernation in comparison with hibernating bats: (A) *Staphylococcaceae*, (B) *Yersiniaceae*, (C) *Hafniaceae*, (D) *Enterobacteriaceae*, (E) *Lactobacillaceae*, and (F) *Morganellaceae*. *Q*-values were calculated with MaAsLin2 linear model (LM) analysis following Benjamini–Hochberg false discovery rate correction. Figure S7. Box plots illustrating the abundance of identified orders in the form of colony-forming units per gram (CFU/g) that differ significantly in active bats before and after hibernation in comparison with hibernating bats: (A) Bacilales, (B) Enterobacterales, and (C) Lactobacillales. *Q*-values were calculated with MaAsLin2 linear model (LM) analysis following Benjamini–Hochberg false discovery rate correction.
