# Supplementary material for: Cultivable Gut Microbiota in Synanthropic Bats: Shifts of Its Composition and Diversity Associated with Hibernation
Source: Animals (Basel). 2023 Nov 26;13(23):3658. doi: 10.3390/ani13233658 (PMC10705225; doi:10.3390/ani13233658)
Supplement: Supplementary file 1 [file animals-13-03658-s001.zip › animals-2674458-supplementary.pdf]

Supplementary figures

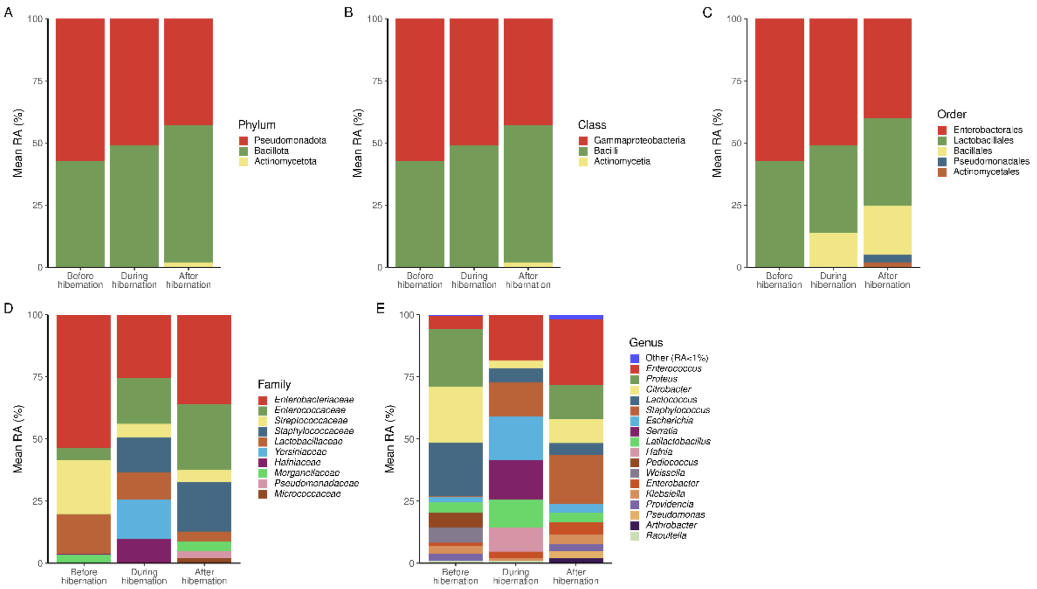

Figure S1. Stacked bar charts representing cultivable gut microbiota composition (as relative abundance [RA] of bacterial taxa) in fecal samples obtained before, during, and after the hibernation of *Nyctalus noctula***.** RA at (A) phylum, (B) class, (C) order, (D) family, and (E) genus, levels. Taxa in the legends are ordered from the most abundant to the least among all three studied groups.

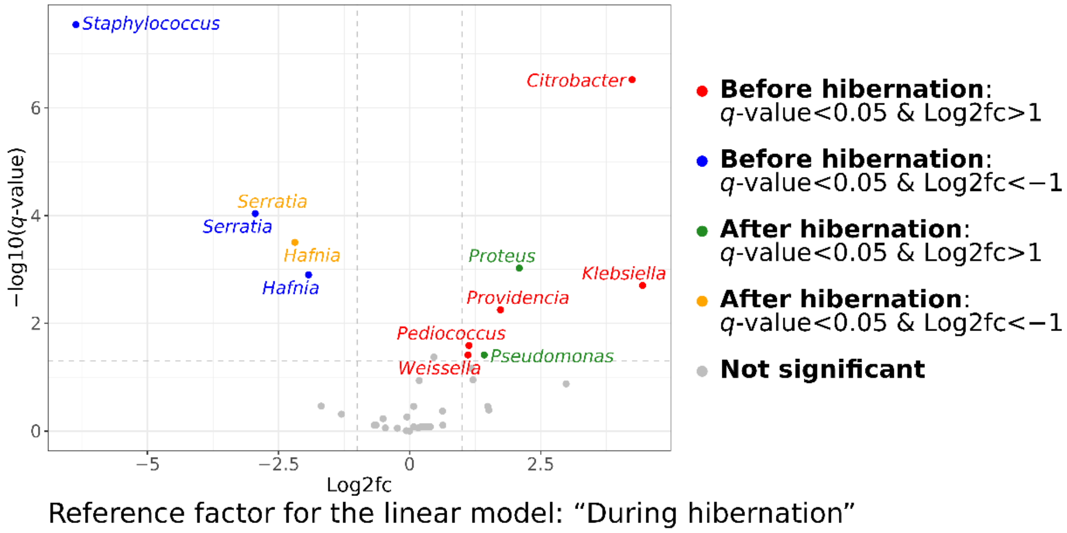

Figure S2. Volcano plot illustrating the results of the differential abundance analysis of bacterial genera isolated from fecal samples of *Nyctalus noctula* before, during, and after hibernation. Microbial composition during hibernation was used as a reference for the linear model analysis. Blue, yellow, green and red dots on the plot show bacteria, the abundance of which had significant associations with studied physiological statuses related to hibernation. Blue dots represent bacteria whose abundance was significantly lower in bats before hibernation in comparison to the reference. Green dots represent bacteria whose abundance was significantly higher in bats after hibernation in comparison to the reference. Yellow dots represent bacteria whose abundance was significantly lower in bats after hibernation in comparison to the reference. Red dots represent bacteria whose abundance was significantly higher in bats before hibernation in comparison to the reference.

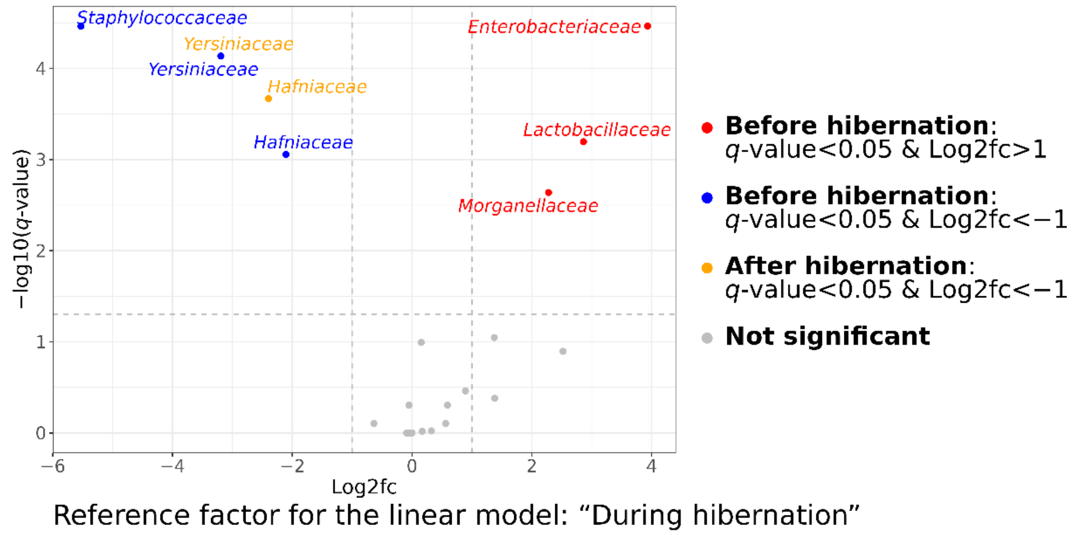

Figure S3. Volcano plot illustrating the results of the differential abundance analysis of bacterial families isolated from fecal samples of *Nyctalus noctula* before, during, and after hibernation. Microbial composition during hibernation was used as a reference for the linear model analysis. Blue, yellow, and red dots on the plot show bacteria, the abundance of which had significant associations with studied physiological statuses related to hibernation. Blue dots represent bacteria whose abundance was significantly lower in bats before hibernation in comparison to the reference. Yellow dots represent bacteria whose abundance was significantly lower in bats after hibernation in comparison to the reference. Red dots represent bacteria whose abundance was significantly higher in bats before hibernation in comparison to the reference.

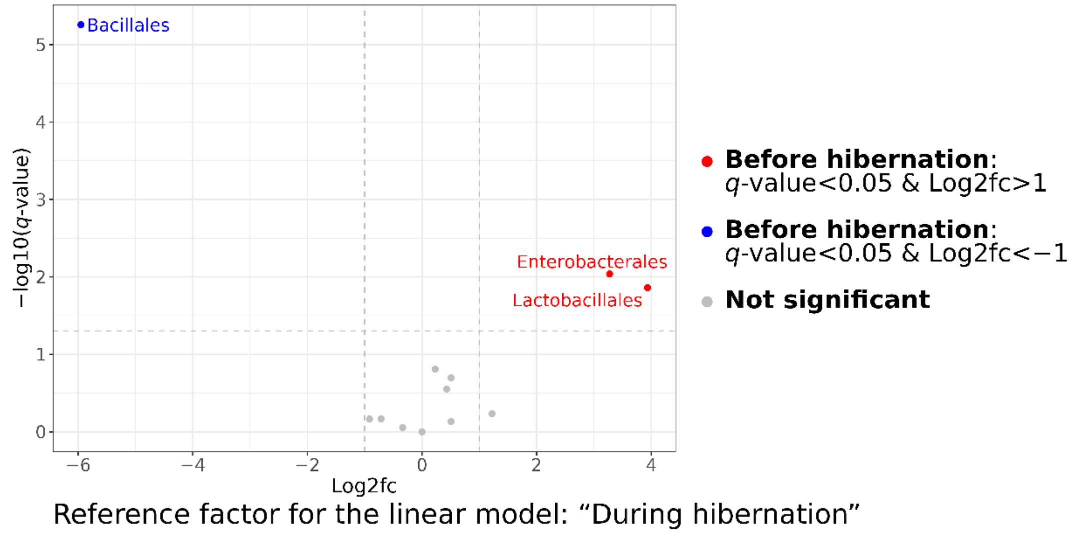

Figure S4. Volcano plot illustrating the results of the differential abundance analysis of bacterial orders isolated from fecal samples of *Nyctalus noctula* before, during, and after hibernation. Microbial composition during hibernation was used as a reference for the linear model analysis. Blue and red dots on the plot show bacteria, the abundance of which had significant associations with studied physiological statuses related to hibernation. Blue dots represent bacteria whose abundance was significantly lower in bats before hibernation in comparison to the reference. Red dots represent bacteria whose abundance was significantly higher in bats before hibernation in comparison to the reference.

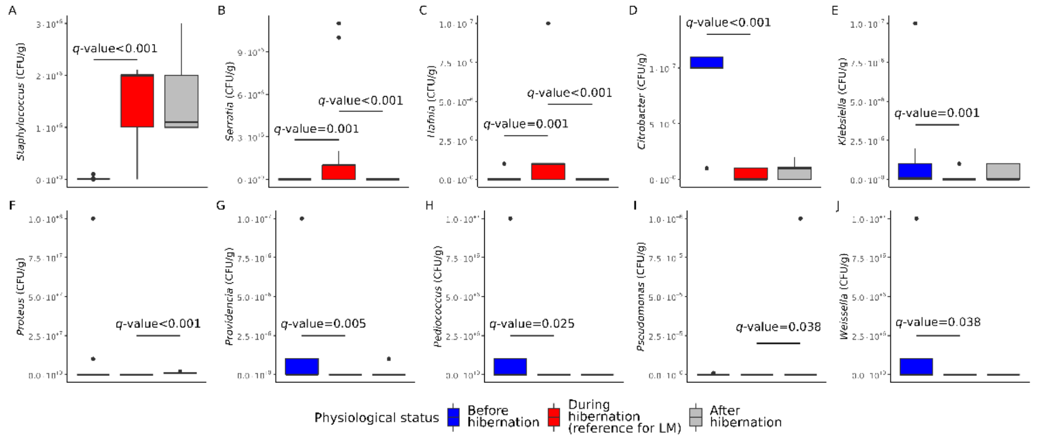

Figure S5. Box plots illustrating the abundance of identified genera in the form of colony-forming unit per gram (CFU/g), that differ significantly in active bats before and after hibernation in comparison to hibernating bats: (A) *Staphylococcus*, (B) *Serratia*, (C) *Hafnia*, (D) *Citrobacter*, (E) *Klebsiella*, (F) *Proteus*, (G) *Providencia*, (H) *Pediococcus*, (I) *Pseudomonas*, (J) *Weisella*. *Q*-values were calculated with MaAsLin2 linear model (LM) analysis following Benjamini-Hochberg false discovery rate correction.

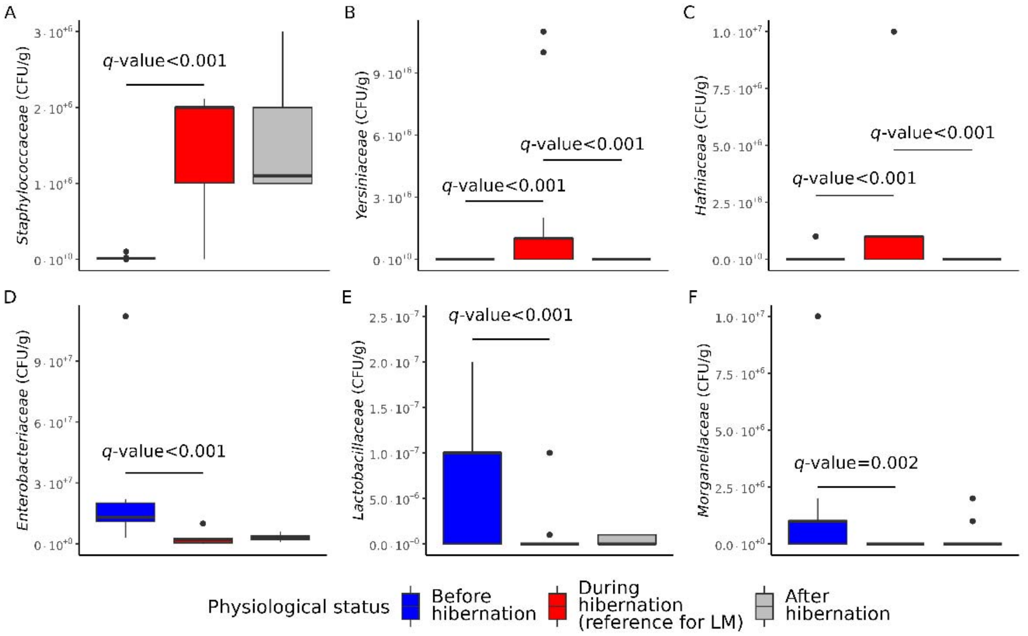

Figure S6. Box plots illustrating the abundance of identified families in the form of colony-forming unit per gram (CFU/g), that differ significantly in active bats before and after hibernation in comparison to hibernating bats: (A) *Staphylococcaceae*, (B) *Yersiniaceae*, (C) *Hafniaceae*, (D) *Enterobacteriaceae*, (E) *Lactobacillaceae*, (F) *Morganellaceae*. *Q*-values were calculated with MaAsLin2 linear model (LM) analysis following Benjamini-Hochberg false discovery rate correction.

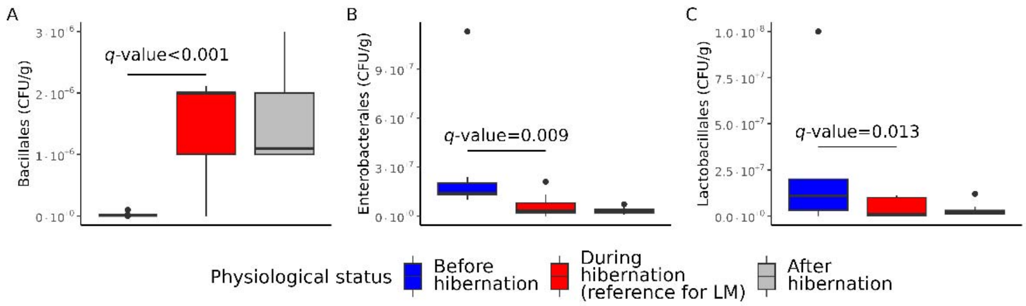

Figure S7. Box plots illustrating the abundance of identified orders in the form of colony-forming unit per gram (CFU/g), that differ significantly in active bats before and after hibernation in comparison to hibernating bats: (A) Bacilales, (B) Enterobacterales, (C) Lactobacillales. *Q*-values were calculated with MaAsLin2 linear model (LM) analysis following Benjamini-Hochberg false discovery rate correction.
